# The Significance of Soy Protein and Soy Bioactive Compounds in the Prophylaxis and Treatment of Osteoporosis

**DOI:** 10.4061/2010/891058

**Published:** 2010-03-08

**Authors:** Sa'eed Bawa

**Affiliations:** Department of Dietetics, Faculty of Human Nutrition and Consumer Sciences, Warsaw University of Life Sciences, Nowoursynowska Street 159C, 02776 Warsaw, Poland

## Abstract

Osteoporosis is defined as a progressive systemic skeletal disease characterized by low bone mass and microarchitectural deterioration of bone tissue, with a consequent increase in bone fragility and susceptibility to fracture. Although bone mass and quality is mainly determined genetically, many other factors, including lifestyle and nutrition also have an impact on bone health. It has been suggested that dietary protein intake may be a risk factor for osteoporosis, and high-protein diets are associated with increased bone loss. Many scientists have examined the relationship between types of protein and urinary calcium excretion, and found that although animal protein was associated with increased urinary calcium excretion, soy protein was not. There is sufficient evidence suggesting soy isoflavones may have potential benefits for bone. Soy protein with naturally occurring phytoestrogens, mainly isoflavones protect against bone loss and synthetic soy ipriflavone in some studies has been shown to favorably affect, but a cause and effect relationship has not been established between the consumption of ipriflavone and maintenance of bone mineral density in post-menopausal women. Therefore it is too early to recommend it as a supplement for this group of women.

## 1. Introduction

Osteoporosis is a skeletal disorder characterized by compromised bone strength, a disorder that predisposes to fractures resulting from no identifiable injury or from minimal trauma insufficient to fracture normal bone. Bone strength is the result of two major determinants: bone mineral content and bone quality. A person's bone mineral content is a function of the interaction of two key factors: the uppermost amount of bone achieved during youth (called the peak bone mass, PBM) in combination with the rate of subsequent bone loss. Growth in bone size and strength occurs during childhood, through adolescence and is usually completed in the 20 s. 

After peak bone mass is achieved during the third decade of life, bone architecture is maintained by a constant remodeling process. Osteoclasts attach to a specific area of bone, remove old bone (resorption pit), then osteoblasts move in and fill this pit with new bone. The balance between these processes shifts at menopause [1] and women typically undergo a rapid phase of bone loss that begins approximately 2 to 3 years before the cessation of menses and continues for up to 5 years postmenopause [2]. Although the decreased concentrations of circulating estrogen observed during menopause and the rapid phase of bone loss are chiefly responsible for the process [2], many other factors are associated with increased fracture risk [3]. These factors include prior fragility fracture, advanced age, a family history of osteoporotic fracture, and the use of certain medications [1]. Additional predictors of bone loss and fracture risk in early postmenopause include prolonged low vitamin D and calcium intake and low body weight as well as high consumption of animal protein, inadequate intake of plant protein, soybean isoflavones and n-3 PUFA [1, 4].

Although hormone replacement therapy (HRT) is able to ameliorate the loss of estrogen at menopause and thus address the concerns related to osteoporosis, there remains substantial interest in alternatives, particularly dietary alternatives, to HRT. It is in this regard that phytoestrogens have received considerable interest in relation to bone health. Related to isoflavones in particular is research showing that ipriflavone, a synthetic isoflavone, effectively reduces bone loss in postmenopausal women [5]. Interest in the potential relation between phytoestrogens and osteoporosis risk was generated from observations of significantly lower numbers of hip fractures in Asian women, relative to Caucasian women [6]. Numerous observational studies relating soy intake of pre- and postmenopausal women to BMD have generally reported significant positive associations [7], with only some studies reporting no association [8]. Some studies assessing BMD have found positive effects of soy and soy isoflavones specifically on BMD of the lumbar spine [8]. Studies assessing biomarkers of bone formation and resorption have been less consistent, with some reporting beneficial effects with respect to bone health [9] while others have reported no significant effects [10] or effects too small to be of clinical relevance [11]. 

Selective estrogen receptor modulators (SERMs), for example, raloxifine have been shown to be effective in preventing bone loss or increasing bone mass. SERMs are a group of chemically diverse non-steroidal compounds that bind to and interact with the estrogen receptors. Soy isoflavones, have been characterized as naturally occurring SERMs with similar beneficial effects to raloxifene on bone without its side effects [12]. This paper focuses on the role of soy protein and soy phytoestrogens, especially, isoflavones as well as the synthetic isoflavone—ipriflavone on the prevention and management of osteoporosis.

## 2. Dietary Sources of Isoflavonoids and Their Biopotency

Isoflavonoids from legumes, including genistein 2 and daidzein, are the most studied phytoestrogens. They can exist as glucosides or as aglycones, the glucosides being readily hydrolyzed in the gut to their aglycones. The aglycones are easily transported across intestinal epithelial cells [13]. Although isoflavones are non-steroidal compounds, they are structurally similar to naturally occurring estrogens, synthetic estrogens and anti-estrogens. The structural similarity of phytoestrogens to endogenous estrogens has prompted the hypothesis that phytoestrogens exert hormonal or anti-hormonal effects relevant to the risk of hormone-dependent disease and/or their suitability as a dietary alternative to hormone replacement therapy. 

Isoflavones are found in highest amounts in soybeans and soy foods, although they are also present in other beans and legumes. Soy foods generally contain 1.2–3.3 mg isoflavones/g dry weight, with the precise amount depending on numerous factors, including the type of soy food [14, 15] as well as soybean variety, harvest year and geographical location [14, 15].  Table 1 below shows the dietary sources of isoflavones. 

Studies have estimated typical isoflavone intakes to be 30–50 mg/day for Asians [14, 15] and less than 1 mg/day for postmenopausal women living in the USA [16]. The plasma concentrations of genistein 2 have been found to range from 0.1–10 mM in the Asian population [17]. 

The major isoflavones present in soy foods include genistein and daidzein and, to a lesser extent, glycitein. Genistein and daidzein are derived from genistin and daidzin as the result of the action of gut microflora. They occur in soy conjugated to sugar moieties as glycosides, termed genistin, daidzin and glycitin. Genistein 2 has one-third the potency of estradiol 1 when it interacts with estrogen receptor-*β* (ER*β*), and one thousandth of the potency of estradiol 1 when it interacts with ER*α*. Genistein 2 can induce similar responses in breast, ovarian, endometrial, prostate, vascular, and bone tissues and cell lines as estradiol 1 [18, 19]. 

Although fermentation of soy can reduce the amount of isoflavonoids present by a factor of 2-3 [15, 16], bioavailability of isoflavonoids is higher in fermented products, so urinary excretion rates are similar for people consuming fermented and unfermented products. Even though genistein 2 has relatively low potency compared to estradiol 1, high concentrations in plasma may be sufficient to cause a variety of physiological effects.

## 3. Bone-Modulating Effects of Soy Protein and Soy Isoflavones

Estrogen plays an important role in maintaining bone density by regulating the formation and resorption of bone [20]. Since lower circulating estradiol-1 levels are found during menopause, calcium is lost from the bone into blood plasma, leading to osteoporosis [21]. One of the aims of hormone replacement therapy (HRT) is to prevent or lower the incidence of osteoporosis in postmenopausal women. 

Prior to the turn of the twentieth century it was assumed that estrogens were exclusively produced by animals. However, the principle that plants can also produce estrogen-like molecules was established by 1966 [22]. Now, it is recognized that certain plants and plant products contain phytoestrogens. One group of such compounds reported to have estrogenic activity is the flavonoids [23, 24]. 

Flavonoids are a large chemical class that are formed through the phenylpropanoid-acetate biodemial pathway via chalcone synthase and condensation reactions with malonyl CoA. Isoflavonoids are a subclass of flavonoids, where one phenolic ring has migrated from C-3 to C-2. The isoflavonoids from legumes, including genistein 2 and daidzein, are the most studied phytoestrogens. They can exist as glucosides or as aglycones, the glucosides being readily hydrolyzed in the gut to their aglycones. The aglycones are easily transported across intestinal epithelial cells [13]. Genistein 2 has one-third the potency of estradiol 1 when it interacts with ER*β*, and one thousandth of the potency of estradiol 1 when it interacts with ER*β* as determined by expression of luciferase reporter gene construct in kidney cells that had been cotransfected with ER*α* and ER*β* [25]. Genistein 2 can induce similar responses in breast, ovarian, endometrial, prostate, vascular, and bone tissues and cell lines as estradiol-1 [19, 26]. Genistein 2 can act as an estrogen antagonist in some tissues. 

Soy isoflavones have an influence not only on sex hormone metabolism, but also possess other biological activities including cholesterol-lowering properties, anti-carcinogenic effects and more recently their protective role in bone health [27, 28]. 

Results of many studies on the protective role of soy protein, its isoflavones, or their combination on bone in ovarian hormone deficient models of osteoporosis are inconsistent. In these studies, the rate of bone formation and bone resorption, as assessed by biochemical markers are unchanged, decreased, or increased. Analogous to animal findings, the effect of soy and its isoflavones on bone in humans are also conflicting. Furthermore, it is not clear whether the bone protective effect of soy protein is due to its amino acid composition [29], nonprotein constituents such as isoflavones [30, 31], or a combination of these factors [31]. Animal studies using the ovariectomized rat model performed by Arjmandi et al. [30] showed that the soy diet with isoflavones was more effective in preventing ovariectomy-induced loss of bone density than either the casein or the soy protein, depleted of its isoflavones, diet. Using an osteopenic rat model, Arjmandi et al. [31] demonstrated a slight reversal of the ovarian hormone deficiency-induced loss of bone with soy protein diets with either normal or reduced isoaflvone content. Although animal findings [30, 31], so far, indicate the importance of isoflavones in preserving bone, it is still not clear whether isoflavones can exert similar bone protective effects independently of soy protein. 

Findings by Picherit et al. [32] support the bone protective role of isoflavones independent of soy protein. These authors reported that isoflavones dose-dependently prevented ovariectomy-induced bone loss in a rat model. However, the same group of investigators did not find similar beneficial effects of isoflavones in reversing bone loss in ovariectomized osteopenic rats [33], even though the rate of bone turnover was reduced. Hence, one can speculate that a longer treatment period with isoflavones would have reversed bone loss. Whether the magnitude of effects of isoflavones on bone in these animal studies by Picherit [32, 33] would have been greater had isoflavones been given in conjunction with soy protein still remains to be answered? 

On the other hand, studies investigating the effects of individual soy isoflavones, genistin and daidzin, support the important role of these naturally occurring compounds in bone health regardless of the dietary protein source. For instance, two weeks of a genistin-rich treatment (1.0 mg/day) in lactating ovariectomized rats was effective in maintaining trabecular bone tissue in comparison with ovariectomized control animals [34]. Furthermore, in the same report, genistin stimulated alkaline phosphatase activity of an osteoblast-like cell line, suggesting a positive effect on bone formation. In another study, Fanti et al. [35] reported that genistein (5 mg/kg body weight) maintained both cortical and trabecular bones in ovariectomized rats, and the bone-sparing effect of genistein appeared to be biphasic.

Although it is believed that genistin is the most potent of all the soy isoflavones, study by Picherit et al. [36] reported that daidzin, is more efficient than genistin in preventing the ovariectomy-induced increase in bone turnover and decrease in bone mineral density. Clearly, this demonstrates that there are uncertainties as to which isoflavone plays a more important role in skeletal health. Use of a single isoflavone may not necessarily be the approach to be taken and future studies should address whether the combination of isoflavones exerts a more pronounced effect on bone. 

To study the relation between soy isoflavone intake and bone mineral density (BMD), Greendale et al.[37] analyzed baseline data from the Study of Women's Health Across the Nation, a US community-based cohort study of women aged 42–52 years. Their 1996-1997 analysis included African-American (*n* = 497), Caucasian (*n* = 1,003), Chinese (*n* = 200), and Japanese (*n* = 227) participants. Genistein and daidzein intakes were highly correlated (*r* = 0.98); therefore, analyses were conducted by using genistein. Median intakes of genistein (measured in micrograms/day) by African Americans and Caucasians were too low to pursue relational analyses further. For Chinese and Japanese women, median genistein intakes were 3,511 and 7,151 *μ*g/day, respectively. Ethnic-specific, linear models were used to predict BMD as a function of energy-adjusted tertile of intake, controlled for relevant covariates. For Chinese women, no association between genistein and BMD was found. Premenopausal, but not perimenopausal, Japanese women whose intakes were greater had higher spine and femoral neck BMD. Adjusted mean spinal BMD of those in the highest tertile of intake was 7.7% greater than that of women in the lowest tertile (*P* = .02); femoral neck BMD was 12% greater in the highest versus the lowest tertile (*P* < .0001). 

Most of the studies suggest that Phytoestrogens are somewhat effective in maintaining bone mineral density in postmenopausal women [38, 39]. A double blind placebo controlled study of postmenopausal women showed significant increase in BMD at the femoral neck after 12 months of daily administration of 54 mg genistein, isolated from soy, although a significant increase in osteocalcin and bone specific alkaline phosphatase (BAP) was also observed [39]. In contrast 17*β*-estradiol 1 increased BMD with a significant decrease in osteocalcin and bone alkaline phospatase (BAP) levels [39]. 

In a 24-week study comparing isoflavone rich soy protein (80.4 mg aglycone isoflavones/day) and isoflavone poor soy protein (4.4 mg aglycone isoflavones/day) in perimenopausal women, both BMD and bone mineral content (BMC) were significantly higher with the diet high in isoflavones [38]. There was no significant change in the BMD or BMC of the lumbar spine over the 24 weeks for the women on either the high or low isoflavone soy protein diet; however, the woman on the control diet had a significant decrease of BMD and BMC during this time. The women who had more bone loss had higher BAP levels. Therefore BAP may be a better indicator of bone turnover than bone formation [38]. 

The lumbar spine seems to benefit the most from consumption of soy phytoestrogens. A 24-week long study with postmenopausal women consuming soy protein with 90 mg isoflavones/day showed a significant increase in BMD of the lumbar spine, with no effect on the femoral neck or total body BMD [40]. There were no BMD effects in woman consuming soy protein with 56 mg isoflavones/day [40]. The only study to examine the effects of soy isoflavones in premenopausal women showed no effect on bone mineral density levels [41], while significant effects were observed in postmenopausal women in this study. 

Brink et al. [42] carried out studies in healthy men (59.2 ± 17.6 y), who were assigned to consume 40 g of either SP or milk-based protein (MP) daily for 3 mo in a double-blind, randomized, controlled, parallel design. Serum insulin-like growth factor-I (IGF-I), which is associated with higher rates of bone formation, was greater (*P*< .01) in men supplemented with SP than in those consuming MP. Serum alkaline phosphatase and bone-specific alkaline phosphatase activities, markers of bone formation, and urinary deoxypyridinoline excretion, a specific marker of bone resorption, were not different between the SP and MP groups. Furthermore, because substantial reductions in bone density occur in men at ~65 y of age, these authors analyzed separately data for men ≥65 y and those <65 y of age. The response to protein supplementation was consistent in the two age groups. The effects of SP on serum IGF-I levels suggest that SP may positively influence bone in men. It should however be mentioned that their study was short, so longer-duration studies examining the effects of SP or its isoflavones on bone turnover and bone mineral density and content in men are warranted. 

There are studies showing that the consumption of isoflavones has no any benefits for women in early postmenopausal period. Brink et al. [42] conducted a randomized, double-blind, placebo-controlled, parallel, multicenter trial in two hundred thirty-seven healthy early postmenopausal women (mean (±SD) age of 53 ± 3 y and time since last menses of 33 ± 15 mo), who consumed isoflavone-enriched foods providing a mean daily intake of 110 mg isoflavone aglycones or control products for 1 y while continuing their habitual diet and lifestyle. These authors found that Consumption of isoflavone-enriched products did not alter bone mineral density of the lumbar spine and total body or markers of bone formation and bone resorption. Furthermore, Hormone concentrations did not differ between the isoflavone and control groups. The intake of isoflavone-enriched products resulted in increased isoflavone concentrations in plasma and urine, whereas control products did not. 

A recent meta-analysis by Liu et al. [43] showed only very slight benefits of soy isoflavones in increasing BMD in women. These authors identified 10 eligible RCTs in 896 women. A mean dose of 87 mg soy isoflavones for at least one year did not significantly affect BMD changes. The mean (95% CI) differences in BMD changes (in mg/cm^2^/year) were 4.1 (−1.6, 9.8) (0.4%) at the lumbar spine, −1.5 (−7.2, 4.3) (−0.3%) at the femoral neck under random-effects model, and 2.5 (−0.5, 5.4) (0.2%) at the total hip by fix-effects model, respectively. Similar results were obtained in subgroup analyses by isoflavone sources (soy protein versus isoflavone extract), ethnic differences (Asian versus Western). Larger dose (≥80 mg/d), but not lower dose (<80 mg/d), of isoflavone intervention tended to have a weak beneficial effect on spine BMD (*P* =  .08 versus *P* = .94). Liu et al. [43] concluded that soy isoflavone supplementation is unlikely to have significant favorable on BMD at the lumbar spine and hip in women.

## 4. Isoflavones and Bone Health: Mechanisms of Action

Soy isoflavones are natural products that could be used as an alternative to menopausal hormone therapy because they are structurally and functionally related to 17beta-estradiol. Results of mechanistic studies indicate that soy or its isoflavones, while suppressing the rate of bone resorption concomitantly enhance the rate of bone formation. In ovariectomized rats, soy isoflavones have been shown [32, 33] to reduce the urinary excretion of deoxypyridinoline (Dpd), a specific marker of bone resorption. Similarly, at least three human studies have clearly demonstrated the anti-resorptive properties of soy or its isoflavones. One of these studies was a cross-sectional study by Horiuchi et al. [44], in which soy protein intake in Japanese postmenopausal women was associated with lower urinary Dpd excretion. In the second study, postmenopausal women who were assigned to a soy milk regimen, providing 60–70 mg of isoflavones, experienced a significant decrease in urinary N-terminal cross-linked peptide, another specific marker of bone resorption [45]. Arjmandi et al. [46] reported that the daily consumption of 40 g soy protein, but not milk protein, for three months by postmenopausal women who were not on HRT significantly reduced the urinary excretion of Dpd. 

Ma et al. [47] and Choi et al. [48] carried out a meta-analysis of nine studies with a total of 432 subjects on the impact of isoflavone consumption on bone markers. They found that urinary Dpd concentration in subjects who consumed isoflavones decreased significantly by −2.08 nmol/mmol (95% confidence interval (CI): −3.82 to −0.34 nmol/mmol) in comparison with that in subjects who did not consume isoflavones. Isoflavone intake versus placebo intake significantly increased BAP by 1.48 *μ*g/L (95% CI: 0.22–2.75 *μ*g/L). Decreases in the urinary Dpd concentration with isoflavone intake of <90 mg/day and with treatment lasting less than 12 weeks were −2.34 nmol/mmol (95% CI: −4.46 to −0.22 nmol/mmol) and −2.03 nmol/mmol (95% CI: −3.20 to −0.85 nmol/mmol), respectively. 

It is reasonable to suggest that soy or its isoflavones enhance bone formation based on at least two lines of evidence: (1) soy isoflavones stimulate osteoablastic activity through activation of estrogen receptors [47, 48], and (2) soy or its isoflavones promote insulin-like growth factor-I (IGF-I) production [31]. In a recent review, Atmaca [49] stated that soy isoflavones act on both osteoblasts and osteoclasts through genomic and nongenomic pathways. Using a rat model of osteopenia, Arjmandi et al. [31] found that soy protein increased the gene expression of IGF-I as indicated by higher femoral mRNA levels. In that study, incorporation of soy protein with normal isoflavone content (2.3 mg/g protein) had a greater effect on femoral IGF-I mRNA than the isoflavone-depleted soy protein-based diet (approximately 0.1 mg/g protein). This finding indicates that isoflavones may have a role in enhancing the synthesis of IGF-I and more importantly at the bone level. Similarly, Arjmandi et al. [46] showed that soy protein supplementation also significantly increased serum IGF-I levels in humans. It is well recognized that IGF-I enhances osteoblastic activity in humans and IGF-I concentrations have been reported to correlate positively with bone mass in pre- [50], peri- [51], and post- [52] menopausal women. Nonetheless, these indirect observations should be confirmed using in vitro and in vivo models including long-term human studies in which more definitive techniques such as bone histology and bone histomorphometry are assessed. 

Ma et al. [53] carried out a meta-analysis of 10 randomized controlled trials on the influence of soy isoflavone intake on spine bone mineral density (SBMD) in 608 menopausal women and found that isoflavones contributed significantly to the increase of SBMD, especially in post-menopausal women. They concluded that Intervention duration for 6 months may be enough for isoflavones to produce favorable effects on spinal bone and that the intake of isoflavone at the level of > 90 mg/day may be better for protecting against spinal bone loss.

## 5. Ipriflavone: The Synthetic Isoflavone and Management of Osteoporosis

Ipriflavone (IP) is an isoflavone, synthesized from the soy isoflavone, daidzein. Only a few studies have been conducted with IP to determine its efficacy in inhibiting the decrease in BMD and only six intervention studies have been so far carried out in humans on the effects of IP on bone mass and turnover [54–59]. Other papers related to ipriflavone include a review of randomised controlled trials (RCTs) [60] and a review on the mechanisms of action of iproflavone on bone metabolism [61]. There has been so far only one large multicentre RCT on the effects of ipriflavone on BMD and turnover in postmenopausal women [62]. 

Several in vitro studies suggest that ipriflavone (typically 200 mg orally 3 times per day) may inhibit bone resorption and increase bone formation, a mechanism by which ipriflavone could prevent bone loss in postmenopausal women [61]. Four of the human intervention studies provided assessed the effects of ipriflavone on BMD and biochemical markers of bone turnover in patient populations with acute leukemia [57], with stroke-induced hemiplegia [58], or with pharmacologically-induced hypogonadism caused by the administration a gonadotropin hormone-releasing hormone agonist [54, 59]. It should be mentioned that evidence on the benefits of ipriflavone from these studies does not establish that these patient populations are representative of the general population with regard to bone status, or that results obtained in studies on such patients relating to the treatment of rapid bone loss can be extrapolated to the maintenance of bone mineral density in the general population of adults. 

Three of the papers showing the benefits of ipriflavone report the results of four randomised controlled trials investigating the effects of ipriflavone consumption on BMD and/or bone turnover which have been conducted in postmenopausal women with either osteopenia or osteoporosis. One of the papers [60] reviewed two Italian multicentre studies performed in elderly women with established osteoporosis. Elderly women with diagnosis of osteoporosis and prevalent vertebral fractures were enrolled in seven centres. A total of 149 subjects entered the two studies, and 111 completed the 2-year intervention period. In both studies, the women were randomly allocated to receive either ipriflavone 600 mg/day or placebo for two years according to a double-blind, placebo-controlled, parallel design. In the first study, 14 women in the ipriflavone group and 13 in the placebo completed the 2-year treatment. Radial BMD significantly increased at years one (by 4%) and two (by 5%) of the intervention in the ipriflavone group compared with placebo. The urinary hydroxyproline/creatinine ratio decreased significantly in the ipriflavone group compared to placebo. In the second study, 41 women in the ipriflavone group and 43 in the placebo group completed the 2-year treatment. Radial BMD significantly increased at years one and two of the intervention in the ipriflavone group compared with placebo. The urinary hydroxyproline/creatinine ratio decreased significantly in the ipriflavone group compared to placebo. The methodological weaknesses of these studies include small sample size, no intention-to-treat analysis, and high rate of drop outs in the first study, which all limit the conclusions that can be drawn in relation to the benefits of ipriflavone in preventing the decrease in BMD. 

Gennari [55] randomised 56 postmenopausal women with low vertebral BMD and postmenopausal age less than five years to receive either ipriflavone (200 mg three times daily) or placebo for two years. All subjects received also 1,000 mg/d of calcium. A statistically significant lower decrease in vertebral BMD was observed in the ipriflavone group compared with placebo at one and two years (−0.4% and −4.9% in the iproflavone and placebo groups at two years, resp.). At the end of the study, urine hydroxyproline/creatinine excretion, a marker of bone resorption, was significantly higher in the placebo group than in the ipriflavone group. 

In the study by Ohta et al. [56], 60 women with postmenopausal osteopenia or osteoporosis were randomly assigned to receive either 600 mg/d ipriflavone or 0.8 g/d calcium lactate for one year. The rate of the decrease in L2-4 BMD was significantly greater in the calcium lactate group than in the ipriflavone group. Median urinary deoxypyridinoline concentrations significantly decreased in the ipriflavone group after one year compared to baseline, whereas no changes were observed in the control group. No statistical comparison between ipriflavone and control groups was reported for this marker of bone resorption. 

In a large prospective, randomised, double-blind, placebo-controlled trial including 474 postmenopausal Caucasian women aged 45 to 75 years with osteopenia or osteoporosis Alexandersen et al. [62] randomly assigned subjects to receive either ipriflavone (200 mg 3 times per day, *n* = 234) or placebo (*n* = 240) for three years in addition to 500 mg/d of calcium. Based on an intention-to-treat analysis, the annual percent change from baseline in BMD at the lumbar spine or at any of the other sites measured did not differ significantly between the ipriflavone and the placebo groups after 36 months of treatment. The response of biochemical markers of bone turnover did not differ between groups. The number of women with new vertebral fractures was not different between the intervention and control groups after three years of follow-up. 

Although four small intervention studies suggest a role of ipriflavone in attenuating the loss of BMD at the lumbar spine in postmenopausal women by decreasing bone resorption, the largest RCT with the highest number of subjects and the longest followup indicates that ipriflavone does not prevent bone loss or affect biochemical markers of bone turnover in postmenopausal women. Furthermore, a cause and effect relationship has not been established between the consumption of ipriflavone and maintenance of bone mineral density in postmenopausal women.

## 6. Concluding Remarks

Diet and nutrition contribute to the different rates of cancer and other diseases throughout the world. Diets rich in plant-derived products may supply a variety of phytoestrogens capable of producing a range of pharmacological effects in the human body. As people live longer, women are spending more of their lives in menopause, affected by a variety of estrogen-related conditions such as osteoporosis, cognitive decline and cardiovascular diseases. 

Soy protein has a modest beneficial effect on bone. However, after the analyses of existing literature it should be stated that it is too early to state whether soy protein or its isoflavones can be substituted for estrogen in preventing the bone loss induced by ovarian hormone deficiency. More studies need to be conducted to find out whether: (1) isoflavones independent of soy protein can prevent ovarian hormone deficiency-associated bone loss; (2) consumption of soy containing food or intake of isoflavones on a daily basis is necessary to observe the expected beneficial effects on bone or simply intermittent use will produce the same results; (3) the effect of soy protein or its isoflavones on bone is transitory; (4) the combination of soy isoflavones and lower doses of estrogens can prevent postmenopausal bone mineral loss and at the same time lower the estrogen-associated risks. It is essential that future studies be performed with standardized and structurally characterized mixtures of compounds or with isolated phytoestrogens. Also more research is needed to determine the efficacy of ipriflavone in inhibiting the decrease in BMD and it is too early to recommend it as supplement for patients with osteoporosis.

## Figures and Tables

**Table 1:** Isoflavonoid content of selected legumes and soy-based foods [16].

Food	Genistein 2 (mg/100 g)	Daidzein (mg/100 g)	Total isoflavonoids (mg/100 g)*
Soy-based infant formula	1.6–15	0.8–9.7	2.6–31
Soy milk	1.1–11.3	1.1–9.8	1.3–21
Soybeans, mature	1.1–150	0.5–91	1.7–221
Tofu	5.0–42.1	0.6–25.6	3.6–67.5
Beans	0.007–0.5	0.008–0.04	0.015–0.5
Chick peas	0.07–0.2	0.01–0.2	1.1–3.6

*Including formomonetin and biochanin.

## References

[1] National Osteoporosis Foundation (2003). *Physician’s Guide to Prevention and Treatment of Osteoporosis*.

[2] Eastell R, Favus MJ (2003). Pathogenesis of postmenopausal osteoporosis. *Primer on the Metabolic Bone Diseases and Disorders of Mineral Metabolism*.

[3] Marshall D, Johnell O, Wedel H (1996). Meta-analysis of how well measures of bone mineral density predict occurrence of osteoporotic fractures. *British Medical Journal*.

[4] Picard D, Imbach A, Couturier M, Lepage R, Ste-Marie L-G (2000). Longitudinal study of bone density and its determinants in women in perior early menopause. *Calcified Tissue International*.

[5] Gennari C, Agnusdei D, Crepaldi G (1998). Effect of ipriflavone—a synthetic derivative of natural isoflavones—on bone mass loss in the early years after menopause. *Menopause*.

[6] Lauderdale DS, Jacobsen SJ, Furner SE, Levy PS, Brody JA, Goldberg J (1997). Hip fracture incidence among elderly Asian-American populations. *American Journal of Epidemiology*.

[7] Kritz-Silverstein D, Goodman-Gruen DL (2002). Usual dietary isoflavone intake, bone mineral density, and bone metabolism in post-menopausal women. *Journal of Women’s Health and Gender-Based Medicine*.

[8] Nagata C, Shimizu H, Takami R, Hayashi M, Takeda N, Yasuda K (2002). Soy product intake and serum isoflavonoid and estradiol concentrations in relation to bone mineral density in postmenopausal Japanese women. *Osteoporosis International*.

[9] Scheiber MD, Liu JH, Subbiah MTR, Rebar RW, Setchell KDR (2001). Dietary inclusion of whole soy foods results in significant reductions in clinical risk factors for osteoporosis and cardiovascular disease in normal postmenopausal women. *Menopause*.

[10] Hsu CS, Shen WW, Hsueh YM (2001). Soy isoflavone supplementation in post-menopausal women. Effects on plasma lipids, antioxidant enzyme activities and bone density. *Journal of Reproductive Medicine*.

[11] Wangen KE, Duncan AM, Merz-Demlow BE (2000). Effects of soy isoflavones on markers of bone turnover in premenopausal and postmenopausal women. *Journal of Clinical Endocrinology and Metabolism*.

[12] Setchell KDR (2001). Soy isoflavones—benefits and risks from nature’s selective estrogen receptor modulators (SERMs). *Journal of the American College of Nutrition*.

[13] Dixon RA, Ferreira D (2002). Genistein. *Phytochemistry*.

[14] Wang H-J, Murphy PA (1994). isoflavone content in commercial soybean foods. *Journal of Agricultural and Food Chemistry*.

[15] Wang H-J, Murphy PA (1994). isoflavone composition of American and Japanese soybeans in Iowa: effects of variety, crop year, and location. *Journal of Agricultural and Food Chemistry*.

[16] Cornwell T, Cohick W, Raskin I (2004). Dietary phytoestrogens and health. *Phytochemistry*.

[17] de Kleijn MJ, Van der Schouw YT, Wilson PWF (2001). Intake of dietary phytoestrogens is low in postmenopausal women in the United States: the framingham study. *Journal of Nutrition*.

[18] Adlercreutz H, Markkanen H, Watanabe S (1993). Plasma concentrations of phyto-oestrogens in Japanese men. *The Lancet*.

[19] Zhou S, Turgeman G, Harris SE (2003). Estrogens activate bone morphogenetic protein-2 gene transcription in mouse mesenchymal stem cells. *Molecular Endocrinology*.

[20] Nilsson S, Gustafsson J-A (2002). Biological role of estrogen and estrogen receptors. *Critical Reviews in Biochemistry and Molecular Biology*.

[21] Yamaguchi M (2002). isoflavone and bone metabolism: its cellular mechanism and preventive role in bone loss. *Journal of Health Science*.

[22] Brzezinski A, Debi A (1999). Phytoestrogens: the “natural” selective estrogen receptor modulators?. *European Journal of Obstetrics Gynecology and Reproductive Biology*.

[23] Riddle JM, Estes JW (1992). Oral contraceptives in ancient and medieval times. *American Scientist*.

[24] Miksicek RJ (1993). Commonly occurring plant flavonoids have estrogenic activity. *Molecular Pharmacology*.

[25] Kuiper GGJM, Lemmen JG, Carlsson B (1998). Interaction of estrogenic chemicals and phytoestrogens with estrogen receptor *β*. *Endocrinology*.

[26] Wang D, Gutkowska J, Marcinkiewicz M, Rachelska G, Jankowski M (2003). Genistein supplementation stimulates the oxytocin system in the aorta of ovariectomized rats. *Cardiovascular Research*.

[27] Ho SC, Chan SG, Yi Q, Wong E, Leung PC (2001). Soy intake and the maintenance of peak bone mass in Hong Kong Chinese women. *Journal of Bone and Mineral Research*.

[28] Wangen KE, Duncan AM, Merz-Demlow BE (2000). Effects of soy isoflavones on markers of bone turnover in premenopausal and postmenopausal women. *Journal of Clinical Endocrinology and Metabolism*.

[29] Omi N, Aoi S, Murata K, Ezawa I (1994). Evaluation of the effect of soybean milk and soybean milk peptide on bone metabolism in the rat model with ovariectomized osteoporosis.. *Journal of Nutritional Science and Vitaminology*.

[30] Arjmandi BH, Birnbaum R, Goyal NV (1998). Bone-sparing effect of soy protein in ovarian hormone-deficient rats is related to its isoflavone content. *American Journal of Clinical Nutrition*.

[31] Arjmandi BH, Getlinger MJ, Goyal NV (1998). The role of soy protein with normal or reduced isoflavone content in reversing bone loss induced by ovarian hormone deficiency in rats. *American Journal of Clinical Nutrition*.

[32] Picherit C, Chanteranne B, Bennetau-Pelissero C (2001). Dose-dependent bone-sparing effects of dietary isoflavones in the ovariectomised rat. *British Journal of Nutrition*.

[33] Picherit C, Bennetau-Pelissero C, Chanteranne B (2001). Soybean isoflavones dose-dependently reduce bone turnover but do not reverse established osteopenia in adult ovariectomized rats. *Journal of Nutrition*.

[34] Anderson JJB, Ambrose WW, Garner SC (1998). Biphasic effects of genistein on bone tissue in the ovariectomized, lactating rat model. *Proceedings of the Society for Experimental Biology and Medicine*.

[35] Fanti P, Monier-Faugere MC, Geng Z (1998). The phytoestrogen genistein reduces bone loss in short-term ovariectomized rats. *Osteoporosis International*.

[36] Picherit C, Coxam V, Bennetau-Pelissero C (2000). Daidzein is more efficient than genistein in preventing ovariectomy-induced bone loss in rats. *Journal of Nutrition*.

[37] Greendale GA, FitzGerald G, Huang M-H (2002). Dietary soy isoflavones and bone mineral density: results from the Study of Women’s Health Across the Nation. *American Journal of Epidemiology*.

[38] Alekel DL, Germain AS, Peterson CT, Hanson KB, Stewart JW, Toda T (2000). isoflavone-rich soy protein isolate attenuates bone loss in the lumbar spine of perimenopausal women. *American Journal of Clinical Nutrition*.

[39] Morabito N, Crisafulli A, Vergara C (2002). Effects of genistein and hormone-replacement therapy on bone loss in early postmenopausal women: a randomized double-blind placebo-controlled study. *Journal of Bone and Mineral Research*.

[40] Potter SM, Baum JA, Teng H, Stillman RJ, Shay NF, Erdman JW (1998). Soy protein and isoflavones: their effects on blood lipids and bone density in postmenopausal women. *American Journal of Clinical Nutrition*.

[41] Mei J, Yeung SSC, Kung AWC (2001). High dietary phytoestrogen intake is associated with higher bone mineral density in postmenopausal but not premenopausal women. *Journal of Clinical Endocrinology and Metabolism*.

[42] Brink E, Coxam V, Robins S, Wahala K, Cassidy A, Branca F (2008). Long-term consumption of isoflavone-enriched foods does not affect bone mineral density, bone metabolism, or hormonal status in early postmenopausal women: a randomized, double-blind, placebo controlled study. *American Journal of Clinical Nutrition*.

[43] Liu J, Ho SC, Su Y-X, Chen W-Q, Zhang C-X, Chen Y-M (2009). Effect of long-term intervention of soy isoflavones on bone mineral density in women: a meta-analysis of randomized controlled trials. *Bone*.

[44] Horiuchi T, Onouchi T, Takahashi M, Ito H, Orimo H (2000). Effect of soy protein on bone metabolism in postmenopausal Japanese women. *Osteoporosis International*.

[45] Scheiber MD, Setchell KDR, Liu JH Dietary soy supplementation reduces LDL-oxidation and bone turnover in healthy postmenopausal women.

[46] Arjmandi BH, Khalil DA, Lucas EA (2000). Soy protein with its isoflavones improves bone markers in middle-aged and elderly women. *FASEB Journal*.

[47] Ma D-F, Qin L-Q, Wang P-Y, Katoh R (2008). Soy isoflavone intake inhibits bone resorption and stimulates bone formation in menopausal women: meta-analysis of randomized controlled trials. *European Journal of Clinical Nutrition*.

[48] Choi EM, Suh KS, Kim YS (2001). Soybean ethanol extract increases the function of osteoblastic MC3T3-E1 cells. *Phytochemistry*.

[49] Atmaca A, Kleerekoper M, Bayraktar M, Kucuk O (2008). Soy isoflavones in the management of postmenopausal osteoporosis. *Menopause*.

[50] Romagnoli E, Minisola S, Carnevale V (1993). Effect of estrogen deficiency on IGF-I plasma levels: relationship with bone mineral density in perimenopausal women. *Calcified Tissue International*.

[51] Nasu M, Sugimoto T, Chihara M, Hiraumi M, Kurimoto F, Chihara K (1997). Effect of natural menopause on serum levels of IGF-I and IGF-binding proteins: relationship with bone mineral density and lipid metabolism in perimenopausal women. *European Journal of Endocrinology*.

[52] Boonen S, Lesaffre E, Dequeker J (1996). Relationship between baseline insulin-like growth factor-I (IGF-I) and femoral bone density in women aged over 70 years: potential implications for the prevention of age-related bone loss. *Journal of the American Geriatrics Society*.

[53] Ma D-F, Qin L-Q, Wang P-Y, Katoh R (2008). Soy isoflavone intake increases bone mineral density in the spine of menopausal women: meta-analysis of randomized controlled trials. *Clinical Nutrition*.

[54] Gambacciani M, Spinetti A, Piaggesi L (1994). Ipriflavone prevents the bone mass reduction in premenopausal women treated with gonadotropln hormone-releasing hormone agonists. *Bone and Mineral*.

[55] Gennari C (1998). Effect of ipriflavone—a synthetic derivative of natural isoflavones—on bone mass loss in the early years after menopause. *Menopause*.

[56] Ohta H, Komukai S, Makita K, Masuzawa T, Nozawa S (1999). Effects of 1-year ipriflavone treatment on lumbar bone mineral density and bone metabolic markers in postmenopausal women with low bone mass. *Hormone Research*.

[57] Pagano L, Teofili L, Mele L (1999). Oral ipriflavone (7-isopropoxy-isoflavone) treatment for elderly patients with resistant acute leukemias. *Annals of Oncology*.

[58] Sato Y, Kuno H, Kaji M, Saruwatari N, Oizumi K (1999). Effect of ipriflavone on bone in elderly hemiplegic stroke patients with hypovitaminosis D. *American Journal of Physical Medicine and Rehabilitation*.

[59] Somekawa Y, Chiguchi M, Ishibashi T, Wakana K, Aso T (2001). Efficacy of ipriflavone in preventing adverse effects of leuprolide. *Journal of Clinical Endocrinology and Metabolism*.

[60] Agnusdei D, Bufalino L (1997). Efficacy of ipriflavone in established osteoporosis and long-term safety. *Calcified Tissue International*.

[61] Reginster JY (2001). Ipriflavone multicenter european fracture study. Ipriflavone in the treatment of postmenopausal osteoporosis: a randomized controlled trial. *Journal of the American Medical Association*.

[62] Alexandersen P, Toussaint A, Christiansen C (2001). Ipriflavone in the treatment of postmenopausal osteoporosis: a randomized controlled trial. *Journal of the American Medical Association*.

